# The Multifaceted Nature of Bilingualism and Attention

**DOI:** 10.3389/fpsyg.2022.910382

**Published:** 2022-06-03

**Authors:** Ashley Chung-Fat-Yim, Noelia Calvo, John G. Grundy

**Affiliations:** ^1^Department of Communication Sciences and Disorders, Northwestern University, Evanston, IL, United States; ^2^Department of Psychology, York University, Toronto, ON, Canada; ^3^Department of Psychology, Iowa State University, Ames, IA, United States

**Keywords:** bilingualism, attention, executive control, language experience, cognition

## Abstract

Attention has recently been proposed as the mechanism underlying the cognitive effects associated with bilingualism. However, similar to bilingualism, the term attention is complex, dynamic, and can vary from one activity to another. Throughout our daily lives, we use different types of attention that differ in complexity: sustained attention, selective attention, alternating attention, divided attention, and disengagement of attention. The present paper is a focused review summarizing the results from studies that explore the link between bilingualism and attention. For each level of attention, a brief overview of relevant theoretical models will be discussed along with a spotlight on paradigms and tasks used to measure these forms of attention. The findings illustrate that different types and levels of attention are modified by the variety of bilingual experiences. Future studies wishing to examine the effects of bilingualism on attention are encouraged to embrace the complexity and diversity of both constructs rather than making global claims about bilingualism and attention.

## Introduction

The question of whether bilingualism leads to performance benefits on various cognitive measures has been the topic of considerable debate in recent years (Antoniou, 2019). While some studies report that speaking two or more languages improves executive functioning on tasks that recruit inhibition, working memory, and cognitive flexibility (see Bialystok, 2017 for a review), others report null results (e.g., Paap and Greenberg, 2013; Gathercole et al., 2014; von Bastian et al., 2016). Several meta-analyses on bilingualism and cognition have added to the debate with contrasting conclusions, again with some meta-analyses in favor of bilinguals (e.g., Adesope et al., 2010; Grundy and Timmer, 2017; Donnelly et al., 2019; Ware et al., 2020), while others conclude equivalent performance after correcting for publication bias (Lehtonen et al., 2018; Lowe et al., 2021). In a large-scale quantitative Bayesian re-analysis of the studies included in the Donnelly et al. (2019) and Lehtonen et al. (2018) meta-analyses, Grundy (2020) found “decisive” evidence that bilinguals outperform monolinguals far more than expected by chance, even after controlling for sample size and publication bias. This re-analysis was not at odds with the previous meta-analyses as they answered different questions. Rather, the study highlighted the need to determine *when* group differences appear rather than *if* they do. The present review highlights the importance of considering the complexity of different forms of attention when examining the effects of bilingualism on cognition. Bilingualism is also extremely complex and consists of a number of unique experiences. However, most of the field treats attention as a unitary construct and bilingualism as a dichotomous variable rather than embracing the complexity of each. This is problematic because it often leads to failed “replications.” We highlight the need to determine which specific bilingual experiences affect which attentional processes across the lifespan.

Language group differences have often been attributed to the bilingual’s need to direct attention towards the target language, while *ignoring* the non-target language that is co-activated and competing for attention (Marian and Spivey, 2003; review in Kroll et al., 2012). Early proposals included selective attention as the key explanation for how bilingual children excelled in problem-solving tasks compared to monolingual children (Bialystok, 1992, 1999). In the late 1990s and early 2000s, researchers began examining how fluency in a second language influenced one or all three components of executive functioning postulated by Miyake et al. (2000). The components included inhibition (controlled suppression of prepotent responses), working memory (updating and monitoring of mental representations), and shifting (ability to flexibly switch between mental states). Of the three components, inhibition was the most studied based on the assumption that words from the non-target language are suppressed or inhibited (Green, 1998). The inhibitory control model by Green (1998) proposed a supervisory attentional system that tags each lexical representation to a language, such that lexical nodes belonging to the non-target language are then inhibited. However, in a review, Bialystok (2017) noted that an inhibitory account explaining the cognitive outcomes associated with bilingualism is unlikely due to several pieces of evidence.

First, pre-verbal infants raised in bilingual households can correctly anticipate the location of a reward after it has switched locations greater than chance, whereas infants raised in monolingual households perform at chance (Comishen et al., 2019). Pre-verbal infants have yet to produce a language and have only rudimentary representations of either language. The more likely explanation is that the bilingual experience affords bilinguals with a different way to allocate attention to their rich and complex linguistic environment (Bialystok, 2015). Second, in a review of the empirical data across various non-verbal interference tasks, Hilchey and Klein (2011) reported that bilinguals typically outperform monolinguals on *both* congruent and incongruent trials. This is contrary to the inhibitory account which predicts that language group differences would emerge on trials that require conflict and selection (i.e., incongruent trials). Congruent trials do not require inhibition because the distracting or irrelevant information does not produce conflict. In fact, the “distracting” element in congruent trials is often facilitatory, such as in the flanker task (Eriksen and Eriksen, 1974) where the surrounding arrows are pointing in the same direction as the target central arrow. The more likely explanation is that bilinguals are better at adapting to the current task demands regardless of whether the trial is congruent or incongruent by flexibly increasing or decreasing attentional engagement (Hilchey and Klein, 2011; Zhou and Krott, 2018). Third, inhibition is not a unitary construct. On tasks that require withholding a prepotent response (Martin-Rhee and Bialystok, 2008), delaying gratification (Carlson and Meltzoff, 2008; Barac et al., 2016), or controlling impulses (Carlson and Meltzoff, 2008), which are also considered to reflect inhibition, monolinguals and bilinguals perform equivalently. Hence, models based on inhibition alone cannot fully explain the research on bilingualism and cognition. For these reasons, Bialystok (2015, 2017) and more recently Bialystok and Craik (2022) proposed attention as a possible mechanism accounting for the processing differences between monolinguals and bilinguals on non-verbal cognitive tasks.

Similarly, D’Souza et al. (2020, 2021) argued that bilingualism may alter attentional processes because bilinguals are exposed to speech that is varied and less predictable than monolinguals. As bilingually-raised infants divide their time across multiple languages, they receive less input from each of their languages than monolingually-raised infants. In addition, bilingual parents are sometimes themselves in the process of learning the community language and may be providing their infants with less accurate input. As such, bilinguals could potentially be redirecting their attention earlier to less familiar input, leading to longer exploration phases and a preference for novelty. The explanation may shed light on why bilinguals show earlier N2 and P3 components than monolinguals in EEG studies (e.g., Chung-Fat-Yim et al., 2021; Grundy and Chung-Fat-Yim, in press, for a review), take longer to initiate a response but are faster and more efficient at executing a response to the correct location in mouse-tracking studies (Incera and McLennan, 2016, 2018; Damian et al., 2018), and are faster to detect a change than monolinguals on eye-tracking studies (e.g., Kovács and Mehler, 2009).

**Figure 1:**
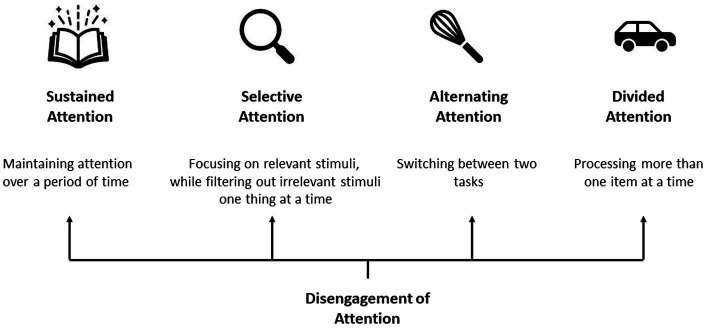
Types of attention and their descriptions.

However, bilingualism is not a monolithic variable and these patterns differ depending on age of acquisition, use, proficiency, context of acquisition, and so on (e.g., DeLuca et al., 2019). Similar to the complexity associated with bilingualism (de Bruin, 2019; Surrain and Luk, 2019), attention also exists along a continuum depending on internal factors (i.e., motivation, prior experience) and external factors (i.e., environmental demands, testing conditions). In fact, the conceptualization of attentional control itself has been debated for decades (Hommel et al., 2019; von Bastian et al., 2020). The definition of attention ranges from “the process of selectively focusing on specific information in the environment” to “directing the mind to an object” or to “the ability to concentrate.” Though these descriptions may sound similar, they recruit attentional resources to varying degrees. The present paper provides a review of the literature on attention and bilingualism by covering different types of attention progressing from low levels of attention to high levels of attention, though these levels can change depending on the task demands: Sustained attention, selective attention, alternating attention, and divided attention (Figure 1). Also, disengagement of attention which underlies all aforementioned forms of attention will be discussed. These forms of attention were chosen based on the different types of attention that have been examined in the literature concerning the effects of bilingualism on cognition. We predict that the largest difference between language groups will emerge on tasks that require greater attentional resources, coinciding with the findings that bilinguals outperform monolinguals when task demands are high (e.g., Qu et al., 2016; Jiao et al., 2019; Comishen and Bialystok, 2021; Kuipers and Westphal, 2021).

Attention is a fluctuating process necessary for concentration when performing a task but also necessary to shift focus, focus on more than one task or avoid distractions (Posner and Boies, 1971; Norman and Shallice, 1986; Hommel et al., 2019; Lindsay, 2020; von Bastian et al., 2020; Wickens, 2021). In general, sustained and selective attention are needed to focus attention on one task at a time, while alternating and divided attention are required for concentration of more than one task. The difference between selective attention and sustained attention is that the former involves focusing on one task while avoiding distractions and the latter refers to a person’s ability to focus on an activity continuously. Alternating and divided attention are both cognitively demanding. While alternating attention refers to switching attention back and forth from one task or stimulus to another, divided attention involves processing multiple tasks or stimuli simultaneously. We were unable to find specific studies on focused attention, which is the ability to concentrate on a stimulus for any given period (even a small duration), thus, it will not be covered in the present review.

## Sustained Attention

From listening to a lecture, reading a book, writing a paper, or watching a movie, sustained attention is crucial to cognitive function and refers to a person’s ability to focus on an activity continuously. Thus, sustained attention is unique in that it involves a duration of a fixed time required to perform an activity (Van Zomeren and Brouwer, 1994; Langner and Eickhoff, 2013). Any momentary lapse in sustained attention due to internal thoughts (e.g., remembering to buy milk while attending a lecture) or external stimuli (e.g., construction noise when trying to read a book) can lead to delays or failure to complete a task. Importantly, these momentary lapses depend on the individual’s motivation or the difficulty of the task to perform. In general, an extended network, including the right frontal and parietal cortical areas (Pardo et al., 1991) together with subcortical areas, are recruited for an unchallenging or repetitive task, while the left hemisphere is additionally recruited for challenging or demanding tasks (Langner and Eickhoff, 2013).

Esterman and Rothlein (2019) recognized five different neurocognitive models of sustained attention, which are based on the related physiological and cognitive functions: arousal, attentional allocation, cognitive control, opportunity costs, and information processing. The terms vigilance, sustained attention, and arousal have been used interchangeably. Moreover, different levels of arousal are related to different attentional mechanisms. In general, arousal is relevant to sustained attention because it is the baseline amount of attentional resources available to perform a task. Esterman and Rothlein (2019) stated, “activity in the locus-coeruleus (LC) noradrenergic system would reduce background noise and enhance neural (phasic) response to salient stimuli, thus enhancing task-related information processing capacity and reducing signal-to-noise-ratios” (p. 175). Also important is how attentional resources are allocated, and the cognitive control processes required for allocation. In brief, arousal would allow the necessary degree of attentional resources, and cognitive control would regulate and allocate the available resources devoted to a task. These mechanisms are affected by the intrinsic cost of control and motivation. In a low arousal state, there would be fewer cognitive resources to be allocated and so task performance may not be optimal, while in a high arousal/distracted state, there would be enough resources, but attention will be less sustained because it is directed towards task-unrelated processes as well (Esterman and Rothlein, 2019).

In the case of bilinguals, sustained attention is required to focus on the target language for a fixed period while suppressing interference from the language that is not being used. Thus, sustained attention is a crucial attentional mechanism that bilinguals first use. However, compared to other forms of attention, research on the relationship between bilingualism and sustained attention is scarce. The evidence thus far indicates that differences between bilinguals and monolinguals in sustained attention are affected by methodological issues or limited to specific tasks. Bialystok et al. (2009) were the first to investigate control processes related to sustained attention in bilinguals. Young and old adult monolinguals and bilinguals were assessed in working memory, lexical retrieval, and cognitive control. Crucially, one of the cognitive control tasks, the Sustained Attention to Response Task (SART; Robertson et al., 1997), included a sustained attention manipulation, in which participants were told to press the spacebar for digits 1 through 9, except for number 3. The next trial appeared after approximately 250 ms, whereas for digit 3, the next trial appeared after 2,000 ms. While the SART showed the typical aging effects, no language group differences were found.

Similarly, Kousaie et al. (2014) compared monolingual and bilingual young adults, as well as monolingual and bilingual older adults on a battery of executive functioning tasks, including the Stroop task (Stroop, 1935), Simon task (Simon and Rudell, 1967), Wisconsin Card Sorting Test (Berg, 1948; Grant and Berg, 1948), digit span subtest of the Wechsler Adult Intelligence Scale (Wechsler, 2008), and the SART, together with a set of language tasks. French-English bilinguals performed better than their monolingual counterparts in Stroop interference, but no language group differences emerged in any of the other executive function measures or language tasks. On the SART, bilinguals were faster than monolingual francophones, but performed equivalently to monolingual anglophones. However, the two monolingual groups were tested in different locations using different equipment for the SART. Thus, the authors attributed this pattern to either technical discrepancies or cultural differences.

The null effects for bilinguals in sustained attention seem to extend to adults with varied ages of L2 acquisition. Bak et al. (2014) evaluated different levels of attention through the “Test of Everyday Attention” (Robertson et al., 1994) in monolingual and bilingual young adults who acquired L2 at different ages. Data consisted of behavioral measures in sustained attention, selective attention, and attentional switching. While the bilingual effects were driven by selective attention and attentional switching, no differences were found between monolingual and bilinguals in sustained attention. These results were replicated in a subsequent study by Vega-Mendoza et al. (2015).

Although these studies found null results in sustained attention between language groups, two important methodological caveats should be considered. First, it is important to note that all of these studies have only reported behavioral or neuropsychological data but no brain measures. It is possible that the behavioral measures in accuracy for young adults reported by Bak et al. (2014) and Vega-Mendoza et al. (2015) were not sensitive enough to detect language group differences in sustained attention. Young adults are at the height of cognitive function, so language group differences may be more difficult to detect with behavioral measures in this age group. Previous research often finds no differences between groups in behavior, but demonstrate less neural activity for bilinguals than monolinguals, indicating efficiency for the bilinguals (e.g., Bialystok et al., 2005; Abutalebi et al., 2012; Grundy et al., 2017b).

Moreover, response time distributions are not gaussian distributions, so statistical analyses of restricted means typically introduce a bias (Miller, 1991; Ratcliff, 1993). Outlier removal which is a common practice to solve the distribution problem brings trial responses closer to the mean. This process reduces group differences and has recently been stated as a crucial problem in bilingualism research (Zhou and Krott, 2016; Grundy, 2020). For instance, consider the study by Martin and Altarriba (2016) who evaluated English monolinguals and English-Chinese bilingual young adults on cognitive control and sustained attention tasks using ex-gaussian analysis to measure behavioral responses. Similar effects were observed for stimulus-response congruency on the Gaussian part of response distributions, but groups differed on the distribution tails showing reduced tails for bilinguals in the more demanding condition. The authors reported that these effects were driven by enhanced sustained attention and attention monitoring.

Finally, the SART is based on a specific aspect of control which is response inhibition (Bunge et al., 2002). As discussed in Bialystok et al. (2009), better tasks to assess sustained attention in bilinguals may be those that also involve selection or interference resolution. Indeed, using a sustained selective attention task combined with EEG, Krizman et al. (2012) found more brain plasticity for bilinguals in subcortical auditory processing. The authors examined the auditory brainstem response to complex sounds (cABR), an index of auditory encoding, through the Integrated Visual and Auditory Continuous Performance Test (Richmond, VA).^1^ For bilinguals, the authors predicted enhanced cABRs to the speech syllable [da] and activation of the fundamental frequency (F0), a feature that underlies pitch perception and is sensitive to experience and perceptual abilities. Results showed that Spanish-English bilinguals had greater subcortical representation of F0, which means that they encoded the stimulus better than English monolinguals, and showed improved sustained selective attention. The authors concluded that through experience-related tuning of attention, the bilingual auditory system is highly efficient in sound processing. Although bilingualism does not appear to influence sustained attention at the behavioral level, using more time-sensitive measures like EEG can capture the precise timing of when these attentional processes diverge between groups.

In sum, several studies have failed to show that bilingualism enhances performance on sustained attention tasks at the behavioral level, but more sophisticated outcome measures (e.g., ex-Gaussian analysis) and neural evidence from an EEG study (Krizman et al., 2012) points to bilingualism enhancing sustained attention. More research is needed to understand what type of bilingual experiences influence sustained attention.

## Selective Attention

We are constantly bombarded with sensory information from the environment. Yet only a fraction of the information spills into our conscious awareness to guide our behaviors. A classic example of selective attention is the “Cocktail Party Effect” (Cherry, 1953), in which individuals can focus on a single conversation despite multiple conversations happening in their surrounding environment. To maintain attention to a single conversation, a filter informs the brain which pieces of information require immediate attention and which ones can be tuned out. This process is known as selective attention. The filter is especially important because attention is a limited resource and attending to multiple things at once can overload the system. Similar to a spotlight in a theater production that shines a light on the characters and/or objects that are central to the plot, our brain directs attention to relevant information in the environment.

One of the most prominent theories of selective attention is stage-like filter theory of Broadbent (1958). In this theory, physical attributes (e.g., color, tone, and pitch) of all incoming sensory information are extracted. A filter then selects what gains conscious awareness and what gets blocked out. Inputs that are selected for further processing are extracted for semantic features and stored in short-term memory, whereas unattended inputs are filtered out and not processed beyond the extraction of physical attributes. Treisman (1964) extended stage-like filter theory of Broadbent (1958) by proposing that irrelevant signals still go through all processing stages, but their signals are attenuated, similar to the volume knob on a stereo. Evidence in support of this claim comes from research with English-French bilinguals, who were presented with a message in English in one ear and the same message in French in the other ear (Treisman, 1964). Despite being instructed to shadow only one ear, participants noticed that the message in the unattended ear was identical to the message in the attended ear. The unattended signal gains conscious awareness if it passes a certain threshold, which is determined by contextual and semantic information. For example, important words, such as our name, have low thresholds. The attenuation theory can account for why individuals are still able to process the meaning of both attended and unattended messages, similar to the co-activation of the non-target language when only a single language is required.

The mechanism underlying selective attention emerges around 4–6 months of age (Johnson and Tucker, 1996; Amso and Johnson, 2008). One of the earliest milestones in infancy is the ability to shift attention from preferentially looking at the eyes towards preferentially looking at the mouth of a speaker. This shift occurs as the infant is learning a new language. Selectively attending to the mouth of a speaker has been shown to predict expressive vocabulary in both monolingual and bilingual infants (Tsang et al., 2018). Infants raised in multilingual households are required to continuously monitor the incoming speech stream to discern one language from the other. Thus, infants raised with two languages have complex linguistic environments and must adapt to their environment by deploying attentional resources differently.

Lewkowicz and Hansen-Tift (2012) speculated that as infants learn to speak, audio-visual cues from speech sounds and lip movements are perceptually salient and useful for imitation. As they begin to master language, a second shift in attention emerges, such that infants divide their attention between the eyes and mouths to evaluate various social cues (e.g., desires and beliefs). Studies have shown that bilingual infants have an earlier start in the attentional shift from the eye region to the mouth (e.g., Pons et al., 2015; Ayneto and Sebastian-Galles, 2017; c.f. Morin-Lessard et al., 2019 for null results). Pons et al. (2015) had 4-, 8-, and 12-month-old Catalan or Spanish monolingual infants and Catalan-Spanish bilingual infants watch native speakers of each language recite a passage in Spanish or Catalan (native languages) or in English (non-native language). While monolingual infants shifted their attention from the speaker’s eyes (4-months of age) to the mouth (8-months of age) to both the eyes and mouth regions in response to the native language only (12-months of age), bilingual infants showed equivalent preference to both the eyes and mouth regions of a speaker at 4- and 8-months of age regardless of the language. The earlier shift in selective attention to the mouth has been observed more in close-language bilinguals (Spanish-Catalan) than distant-language bilinguals (Spanish-“Other”), suggesting that language proximity influences how audiovisual speech cues are evaluated by bilingual infants (Birulés et al., 2018).

Selective attention has also been investigated in infants using the Visual Expectation Cueing Paradigm (VExCP; Baker et al., 2008). In an eye-tracking study, Comishen et al. (2019) compared selective attention processes between 6-month-old infants raised in monolingual households and infants raised in bilingual households on the VExCP paradigm. In this paradigm, a reward can appear in one of two locations. The location of the reward is determined by the presentation of a cue (bullseye or checkerboard). In the pre-switch condition, a cue (i.e., bullseye) predicted the reward on one side of the screen and a different cue (i.e., checkerboard) predicted the reward on the opposite side of the screen. Both groups of participants correctly anticipated the location of the reward. Without any warning, the cue-reward location was switched in the post-switch condition, such that the cues predicted the reward to be on the opposite side of the screen. After the switch, performance of monolingual infants was reduced to chance, but bilingual infants continued to correctly predict the reward’s location. Thus, infants raised in bilingual environments distribute their selective attention more efficiently than monolingual infants and are able to create new associations.

Selective attention differences between monolinguals and bilinguals can also be assessed using the visual search paradigm. The visual search paradigm requires participants to search an array for a particular object (target) amongst multiple objects (distractors). The underlying processes involved when performing a visual search task closely resemble those used to navigate our everyday lives, such as having to find a classmate in a filled lecture hall. Visual search tasks are typically composed of two types of searches: feature and conjunction searches. When the distractors differ from the target by only a single feature, there is a pop-out effect and it is easy to pick out the target from the distractor, this is known as a feature search. In contrast, when the distractors are different from the target by two or more features (e.g., searching for a red triangle among red diamonds and blue triangles), this is known as a conjunction search. For conjunction searches, participants search in a serial manner and use top-down control processes to find the target.

Friesen et al. (2015) compared young adult monolinguals and bilinguals on feature and conjunction searches. For conjunction searches, the authors manipulated discriminability by making the distractors similar in color to the target. Bilinguals outperformed monolinguals only on the most difficult condition (low discriminability, conjunction search; c.f. Paap et al., 2018 for contradictory findings). Similarly, Hernández et al. (2012) compared Catalan-Spanish bilinguals and Spanish monolinguals on three visual search conditions that varied in the recruitment of bottom-up and top-down processes. Bilinguals were faster across all conditions and less impacted by the irrelevant information that was maintained in working memory than monolinguals. Both studies show that bilingualism aids in the development of efficient and effective search strategies, specifically when executing top-down processes.

When searching for a target in a display, top-down processes guide eye-movements through the use of contextual and semantic cues from the environment. In an eye-tracking study, Chabal et al. (2015) had monolingual and bilingual young adults perform a multi-modal visual search task. Participants were first presented auditorily with the name of a target object (e.g., dog). A display of eight objects, including the image of the target object, then appeared along with an auditory sound that could be related (e.g., dog barking) or unrelated (e.g., piano keys) to the target object. A unique feature of the visual search paradigm implemented by Chabal and colleagues is that the objects within the search array were meaningful objects rather than shapes, and were visually different from each other, a scenario that is similar to what is experienced in natural environments. The authors found that bilinguals made more fixations to the target and fewer fixations to the distractor, while monolinguals made the same number of fixations to the target and distractors. Therefore, on visual search tasks, monolinguals and bilinguals employ different search strategies, such that bilinguals are more efficient at locating the target than monolinguals. The combined behavioral and eye-tracking findings provide greater insight into how each language group allocates attentional resources and scans their environment. These findings suggest bilingualism provides a boost on more demanding tasks, such as in conjunction searches, and not on feature searches that involve simple detection.

The ambiguous figures task allows for an examination of selective attention abilities given that the task requires participants to selectively attend to the relevant features of an alternative interpretation in order to see the alternate image during the task. Chung-Fat-Yim et al. (2017) presented young adult monolinguals and bilinguals with an unambiguous image that gradually changed to another unambiguous image. Participants had to name the alternate image using the fewest number of cards. The cards in the middle of the spectrum were ambiguous figures, which are optical illusions that produce different perceptions depending on the perceiver’s focal point. Bilingual young adults required fewer cards to see the alternative image than monolingual young adults, suggesting that they were able to come to a single interpretation from a myriad of other potential interpretations and focus on the relevant features of the alternate image.

In sum, several studies across the lifespan using different types of paradigms have shown that bilingualism enhances selective attention. In fact, the effects of bilingualism on selective attention can be detected quite early (as early as 6 months of age), such that infants who are raised in a bilingual household show greater attentional control to stimuli in their surroundings than those raised in a monolingual household. Hence, knowing more than one language can expand the mind to perceive and interpret problems, objects, and concepts in more ways than one. As a consequence of being raised in a more linguistically complex environment, do multilingual speakers shift attention from one stimulus to another more readily than monolingual speakers?

## Alternating Attention

Alternating attention refers to the rapid shifting of attentional focus due to the inability to process all available information in parallel (Parasuraman, 2000). This includes activities such as reading a book and stopping to answer a phone call, then returning to read the book. According to the model proposed by Posner and Petersen (1990), alternating attention depends on the “orienting network,” which is responsible for directing attention to a target stimulus. Parietal regions and the frontal eye fields have been associated with the orienting network (Fan et al., 2005; Vernet et al., 2014), but the basal ganglia and the cerebellum have also been implicated (Ravizza and Ivry, 2001; Ravizza and Ciranni, 2002).

Alternating attention may be involved in bilingual processing due to the need to shift attention between languages. A common task used to measure alternating attention is the Trail Making Test (TMT). Bialystok (2010) reported evidence showing better performance for bilingual children than monolingual children in the TMT. Crucially, this ability to alternate or shift attention is present from infancy (Posner and Raichle, 1994) and develops with age (Trick and Enns, 1998). However, the evidence for bilingual adults using the TMT is scarce and remains unclear. Goral et al. (2015) assessed alternating attention with the TMT, inhibition with the Simon task, and working memory with the Month Ordering Task. Bilingual older adults, who were either dominant bilinguals or balanced bilinguals, were recruited. The authors found that bilingual type (balanced vs. dominant) predicted performance on the inhibitory control task, but not the working memory task. Later, Estanga et al. (2017) examined cognitive performance on the TMT task while measuring cerebrospinal fluid (CSF) AD-biomarkers amongst monolinguals, early bilinguals, and late bilinguals. Only early bilingualism was associated with lower CSF total-tau. CSF did not interact with the TMT performance, but late bilinguals showed better performance than monolinguals on this task, suggesting enhanced alternating attention for this group.

Similarly, those studies which have assessed task switching in bilinguals generally report different effects in samples containing only young adults. Task switching is relevant to bilingualism because the processes recruited by these tasks are similar to the processes bilinguals engage in code switching and language switching. Using a non-verbal switching paradigm, Wiseheart et al. (2016) compared bilingual and monolingual young adults to investigate the transfer of language switching skills to domain-general task switching. While monolinguals and bilinguals performed similarly when switching between tasks in a mixed block (local switch cost), bilinguals had a reduced mixing cost than monolinguals when comparing performance on the mixed block to the pure block (global switch cost). The authors concluded that using multiple languages leads to more flexibility in task switching due to the attentional mechanisms and cognitive control processes related to this task. Prior and Gollan (2011) used a task switching paradigm that included a non-verbal switching task and a language switching task to evaluate the performance of young adults who were either bilingual or monolingual. Spanish-English bilinguals who reported switching between languages frequently had smaller task switching costs than Mandarin-English bilinguals who reported switching between languages less frequently and monolinguals in both switching paradigms. The bilinguals who switched less frequently performed similarly to monolinguals.

Yim and Bialystok (2012) investigated the relationship between code switching frequency and performance in a verbal and non-verbal task switching paradigm. Cantonese-English bilingual young adults completed a non-verbal code switching paradigm together with a verbal fluency task that required language switching. The authors found that those participants who engaged in more conversational code switching had reduced costs in verbal task switching than those who switched languages less frequently. The non-verbal switching task showed similar results to those reported in previous studies but in this case, performance was not associated with the degree of conversational code-switching. The authors concluded that there might be a dissociation between verbal and non-verbal processes related to cognitive control for the mechanism of task switching. Interestingly, highly proficient bilinguals may have comparable switch costs in both directions when switching languages (L1 and L2), which is known as the “symmetrical cost switch,” and this process may also be more sensitive to verbal tasks. Calabria et al. (2012) tested the symmetrical cost switch hypothesis in young adults who were highly proficient in Catalan (L1), Spanish (L2), and had a low proficiency in English (L3). All participants completed both a linguistic switching task and a non-linguistic one. The results revealed in this case that highly proficient bilinguals had symmetrical switch costs in the linguistic task but not in the non-linguistic task. However, it is important to note that these effects may be affected by the properties of the task (e.g., cue size). Stasenko et al. (2017) evaluated Spanish-English bilinguals and English monolinguals using the shape-color switching task and an analogous language switching task with varying cue-target intervals (CTI, long vs. short) in both tasks. Overall, with longer CTI bilinguals revealed significantly reduced task switching costs than monolinguals, but this was only seen in the first half of the trials as practice benefited RTs on short CTI trials.

Task switching may also involve other mechanisms affecting outcomes beyond alternating attention. In an fMRI study, Weissberger et al. (2015) tested Spanish-English bilingual adults on both a non-linguistic and a language switching paradigm. While there were no differences between tasks on single and switch trials, there were task differences in the repeat trials in the mixed block together with more widespread activation for the non-linguistic switching task. Thus, the authors concluded that the cognitive benefits associated with bilingualism may not be related to switching or alternating between tasks but instead to the joint activation of the networks needed to sustain inhibition. Interestingly, recent research with infants has shown that infants exposed to a bilingual environment are better at shifting attention to a novel stimulus and alternate attention more frequently than infants exposed to a monolingual environment (D’Souza et al., 2020). These early adaptations to the attentional system during infancy have been found to persist into adulthood (D’Souza et al., 2021). Hence, bilinguals have an edge in situations requiring flexibly switching attention between tasks to meet the demands of their rapidly changing environment.

In sum, different bilingual experiences, including balance of first and second languages, code switching frequency, proficiency, and age of acquisition, all influence performance on tasks measuring alternating attention, and these experiences interact with task parameters.

## Divided Attention

Divided attention is the ability to process two or more pieces of information simultaneously. For example, talking on the phone while driving, or doing data analysis while singing along to your favorite song. Researchers sometimes argue that true divided attention is difficult if not impossible for people to do because of a bottleneck at the response-selection stage, during which a response to the first task must be selected before processing begins at the response-selection stage of the second task (Pashler, 1984, 1994; Pashler and Johnston, 1998). This view continues to influence current research (review in Koch et al., 2018), but others have provided evidence that true divided attention can be achieved when response-selection stages overlap between task 1 and task 2 (Watter and Logan, 2006; Thomson et al., 2010; Koob et al., 2021). For example, using a psychological refractory period paradigm (Telford, 1931), Watter and Logan (2006) showed that task 1 response times were faster when task 2 required the same button press as task 1. This demonstrates that the response selection stage for task 2 must have begun prior to the completion of task 1’s response selection stage, otherwise, no priming would occur from task 2 to task 1. It is possible that bilingualism modifies divided attention processes. For instance, unbalanced bilinguals speaking in their second language might simultaneously prime their second language representations (task 1) and their first language representations, which may be automatically primed (task 2).

Very few studies have examined the influence of bilingualism on divided attention. Bialystok et al. (2006) had monolingual and bilingual younger and older adults perform two simultaneous classification tasks. Participants had to determine whether a stimulus, auditorily or visually-presented was: (1) a string of letters or digits and (2) an animal sound or musical instrument. They were also instructed to prioritize the visual modality. Younger and older adult bilinguals were more efficient at categorizing visual stimuli than their monolingual counterparts, suggesting enhanced divided attention. In another study, participants classified objects as either human-made or natural and words as concrete or abstract based on a cue provided to examine non-verbal divided attention. Brito et al. (2016) found that simultaneous bilinguals (i.e., acquired both languages before the age of 5) made fewer errors than monolinguals on switch trials. Sequential bilinguals (i.e., learned a second language after the first language but before the age of 15) did not differ significantly from the other two groups, suggesting that only certain bilingual experiences can lead to enhanced divided attention compared to monolinguals. Fernandes et al. (2007) tested younger and older monolingual and bilingual adults on a verbal divided attention task. The primary task involved memorizing a list of words presented auditorily for a subsequent memory test. In the full attention condition, the lists of words were presented without any distractions. However, in the divided attention condition, a secondary task was administered concurrently with the encoding task, in which participants judged whether visually-presented words were smaller or larger in size than a referent object (e.g., “monitor,” “CPU,” “mouse,” or “keyboard”). In contrast to the authors’ predictions, bilinguals recalled fewer words than monolinguals in the full and divided attention conditions. These findings may be due to the type of task used. Because this task involved encoding and retrieving *verbal* information, bilinguals may have been at a disadvantage considering they hold on average a smaller vocabulary (Bialystok et al., in press) and are slower to retrieve words (e.g., Gollan et al., 2005; Ivanova and Costa, 2008) in each of their languages compared to their monolingual counterparts. In other words, the bilinguals’ cognitive system may have already been taxed from having to remember the verbal information, and with the additional attentional demands required to perform the task, their cognitive resources may have been depleted much more rapidly. Hence, it is important to consider not only the type of attentional process being measured, but also the domain (verbal or non-verbal) under examination.

In sum, the limited evidence suggests that bilingualism may influence divided attention processes, but that these benefits depend on different bilingual experiences (e.g., only for simultaneous and not sequential bilinguals) and different task parameters (e.g., only non-verbal tasks).

## Disengagement of Attention

The ability to engage, disengage, and then re-engage attention on an object of interest is a critical process involved in all of the aforementioned forms of attention. In order to shift attention from one location to another, Posner et al. (1982) argued that attention must first disengage from its current location, move to a new location, and finally fixate on the new location. Critically, disengagement of attention might also be a process enhanced by bilingualism (Mishra et al., 2012; Grundy et al., 2017b). Given that bilinguals must continually focus their attention on multiple aspects of linguistic information over the lifespan, it follows that they may have acquired additional practice and become faster at disengaging attention over time from the information that is no longer relevant in order to focus on current task demands. Rapid disengagement from previously-relevant information would help bilinguals perform better on current task demands when the demands switch, but would hinder performance when demands are repeated (Grundy et al., 2017b). Dense-code switching environments, where bilinguals switch languages between and even within sentences (Green and Abutalebi, 2013), would likely promote rapid disengagement of attention from one language in order to engage in another. Training these domain-general processes helps to explain why bilingualism has been shown to have an influence on task switching performance (Prior and MacWhinney, 2010; Hartanto and Yang, 2016; Wiseheart et al., 2016). Evidence in support of bilingualism leading to more rapid disengagement of attention is supported in several studies across the lifespan.

**Figure 2:**
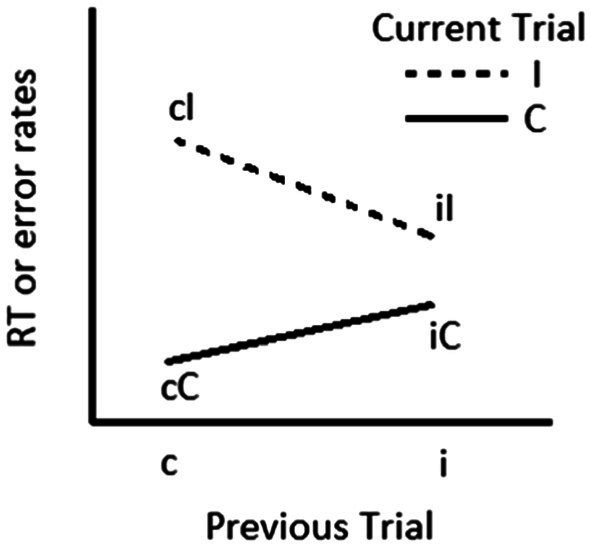
The sequential congruency effect (SCE) is calculated using the following formula: (cI – cC) – (iI – iC). Larger SCEs reflect slower disengagement of attention from previous information on current trial performance. C and c refer to congruent trials. I and i refer to incongruent trials.

Grundy et al. (2017b) demonstrated that bilinguals showed smaller sequential congruency effects (SCEs) than monolinguals on a flanker task, consistent with the interpretation of more rapid disengagement of attention for bilinguals. SCEs, also commonly known as Gratton effects or conflict adaptation effects (Gratton et al., 1992), reflect the finding that individuals show smaller congruency effects (difference in RT or accuracy between incongruent and congruent trials) following incongruent than congruent trials (Figure 2). In essence, SCEs reflect the influence of previous trials on current trial performance and can index the speed at which disengagement occurs. If individuals are slow to disengage attention, they will show larger SCEs, and if individuals are fast to disengage attention, they will show smaller SCEs.

Previous work that has gone unnoticed bolsters the claims by Grundy et al. (2017b showing that bilinguals are likely faster to disengage attention than monolinguals. Using an Attention Network Task (Fan et al., 2002), Costa et al. (2008) examined switch costs between young adult monolinguals and bilinguals. They demonstrated that both groups were slower when responses to congruent trials were preceded by incongruent than congruent trials, but that this “switching cost” was more pronounced for monolinguals than bilinguals. These findings are in line with the SCE findings reported by Grundy et al. (2017b) and consistent with the interpretation that bilinguals disengage attention from previous information more rapidly than monolinguals. More recently, across two experiments, Teubner-Rhodes et al. (2019) showed that trial accuracy decreased for incongruent trials on a Stroop task when preceded by congruent trials (i.e., cI trials) for monolinguals, but not bilinguals. Thus, there is converging evidence that bilinguals show smaller SCEs than monolinguals due to more rapid disengagement of attention.

Some have argued against this position by stating that the group effects for SCEs are not replicable (Goldsmith and Morton, 2018; Paap et al., 2019). Goldsmith and Morton (2018) and Paap et al. (2019) attempted to replicate the patterns observed in the original study by Grundy et al. (2017b) and concluded that the effects were not reliable and that no group differences exist. However, their studies had critical issues with their design features that made the studies non-replications (Grundy and Bialystok, 2019). It is important to note that their studies used long response-to-stimulus intervals (RSI), despite the fact that Grundy and colleagues, in their original 2017 study, clearly argued and demonstrated that group effects were only reliable at shorter (500 ms or less) RSIs. Grundy et al. also showed that when RSIs are long and all individuals have enough time to disengage, the group effects disappear at the behavioral level, but the brain responses show a smaller SCE in the time course for bilinguals than monolinguals. Thus, to date, there is currently only positive evidence for the finding that bilingualism leads to faster disengagement of attention captured by SCEs.

Mishra et al. (2012) provided evidence that within bilinguals, high proficiency bilinguals show a greater inhibition of return effect (IOR) than low proficiency bilinguals and concluded that greater proficiency in a second language leads to more rapid disengagement of attention. The IOR paradigm captures the point at which an irrelevant cue appearing in the target location before presentation of the target becomes inhibitory rather than facilitatory with greater time intervals between cue and target. Earlier and greater IOR effects reflect more rapid disengagement of attention (Posner and Cohen, 1984; Klein, 2000). Saint-Aubin et al. (2018) attempted to replicate the findings by Mishra et al. (2012) study with a different population, but failed to replicate the critical findings. However, without functional brain data, we cannot be certain that the groups were not processing information differently, despite similar behavior. It should also be noted, as previously mentioned, that bilingualism is not a categorical variable (Luk and Bialystok, 2013) and that not all bilingual experiences are the same (de Bruin, 2019); treating them as such risks masking real effects (Grundy, 2020; Leivada et al., 2021). Recent calls in the literature focus on the importance of examining bilingual experiences along a continuum rather than dichotomously.

Grundy et al. (2020) recorded event-related potentials (ERPs) with electroencephalography (EEG) while participants performed the IOR task and showed that there was no difference between low proficiency and high proficiency bilinguals in terms of the IOR effect when examining the groups categorically. Examination of second language proficiency along a continuum revealed a different story—greater second language proficiency reliably predicted greater and earlier IOR effects. Electrophysiological data revealed that disengagement of attention involved multiple cognitive processes across the scalp. Thus, the IOR paradigm provides further evidence that bilingualism leads to more rapid disengagement of attention.

The bivalency effect refers to the slowing that occurs when participants are occasionally presented with a stimulus containing conflicting cues derived from two ongoing tasks. Using a bivalency effect paradigm (e.g., Woodward et al., 2003; Meier et al., 2009; Grundy et al., 2013). Grundy and Keyvani Chahi (2017) showed that bilingual children were less influenced by the appearance of conflicting stimuli while switching between multiple tasks on subsequent non-conflicting stimuli. Grundy and Bialystok (2018) attempted to replicate this pattern in young adults. Considering that young adults are at peak cognitive performance (Hartshorne and Germine, 2015) and behavioral measures often lack the sensitivity to capture subtle differences between groups, the authors tested young adult monolinguals and bilinguals on the bivalency task while EEG was recorded. While bilinguals and monolinguals showed equivalent behavioral performance, event-related potentials demonstrated that monolinguals required greater and longer lasting cognitive processing to handle trials that followed conflict than bilinguals. These findings suggest that younger adult bilinguals are also able to disengage attention more rapidly than monolinguals following conflicting stimuli. Disengagement of attention might contribute to the larger finding that bilinguals are more efficient and faster at processing information on executive function tasks (reviews in Grundy et al., 2017a and Grundy and Chung-Fat-Yim, in press), such that the electrophysiological components associated with attention and conflict monitoring generally appear earlier for bilinguals than monolinguals.

In sum, there is substantial evidence at both the behavioral and neural levels that bilingualism leads to more rapid disengagement of attention from no-longer relevant stimuli. A preliminary experiential contender for more rapid disengagement of attention appears to be greater proficiency in a second language, but other bilingual factors have not yet been explored.

## Conclusion and Future Directions

The present review provides an overview of the complexity involved in understanding research on bilingualism and attention. Both constructs have often been simplified in the literature, and this runs the risk of masking several ways that different bilingual experiences influence different forms of attention. The evidence outlined in the present review highlights some ways in which bilingualism affects different attentional mechanisms.

Bilinguals appear to develop selective attention abilities earlier than monolinguals possibly as a means of facilitating and promoting language acquisition and discrimination. In other words, attentional resources are recruited in bilinguals to allow them to first recognize which speech sound they heard and from which language. The pattern seems to extend to young adults both at the behavioral (e.g., Chabal et al., 2015; Chung-Fat-Yim et al., 2017) and at the brain (Grundy and Chung-Fat-Yim, in press) level.

Alternating attention is less studied, but the initial findings suggest that whether or not bilingualism enhances alternating attention at the behavioral level depends on whether the switching task includes verbal or non-verbal measures. Neuroimaging studies suggest that EF control mechanisms are crucial when alternating attention between tasks and languages. Most of the evidence comes from young adult populations, but a recent study indicates that the effects may also be present during infancy (D’Souza et al., 2021). Similarly, the literature on divided attention is scarce, making it difficult to determine whether bilingualism influences this type of attention. In this case, the results also vary depending on age of participants, type of task, and the verbal/non-verbal distinction. Importantly, all of these types of attention require participants to engage, disengage, and re-engage attention. The most consistent pattern of findings appears in the literature on attentional disengagement demonstrating that bilinguals are faster and more efficient at disengaging from irrelevant information. Although disengagement of attention is crucial in bilingual processing, more research is needed - especially with regards to which experience-based factors modulate attentional processes.

In order to compare findings across studies, it is important to use tasks that have been well-established in the field. However, the vast majority of the research with young adults has used relatively simple executive function (EF) tasks that often yield fast response times and accuracy rates at ceiling with little variability across participants. In addition, such behavioral measures capture only the endpoint of a dynamic chain of attentional processes. While these simple EF tasks should not be fully abandoned, they do need to be re-evaluated in terms of their purpose in addressing the research questions on bilingualism and cognition. Even in instances where the same task was used across studies, modifications are often implemented to the original designs, such as in the proportion of congruent and incongruent trials, the type of stimulus used (e.g., chevrons versus arrows in the flanker task), the experimental design (i.e., whether a neutral block was included in the paradigm as a control condition), the number of breaks administered to participants, and the visual angle of the stimuli, to name a few, all which likely impact EF performance. As the field continues to embrace the complexity associated with bilingualism by placing individuals along a continuum of language-based factors, the present review sought to highlight the complexity associated with the interaction between attention, task/environmental demands, and bilingualism. Future research should strive to design tasks that account for the types of activities performed on a day-to-day basis in more naturalistic settings. Hence, we echo the recommendations made by Poarch and Krott (2019) for researchers to use more ecologically-valid and age-appropriate tasks.

Furthermore, language-based factors of proficiency and usage are often placed at the forefront, whereas other viable language history measures, although collected, are rarely reported. Few studies, for example, report whether the testing session was conducted in the bilingual’s preferred language, despite knowing that this can affect EF outcomes (e.g., Grundy and Timmer, 2017). If the testing session is conducted in the bilingual’s non-native language, the results should be interpreted in light of the language of testing and the participants’ preferred language. By testing participants in the language they are most comfortable with using, participants may perform at optimal levels, as this would minimize the amount of attentional resources devoted towards language processing.

Bilingualism is not a dichotomous variable (Luk and Bialystok, 2013) and the field is starting to recognize the importance of several bilingual experiences affecting neuroplasticity differently (DeLuca et al., 2019; Pliatsikas et al., 2020; Calabria et al., 2021). This is crucial to consider because failed “replications” using groups of “bilinguals” and “monolinguals” may be examining completely different types of bilinguals that would not be expected to show certain types of neuroplasticity. Thus, one should not expect that all bilinguals will outperform all monolinguals on tasks designed to measure different forms of attention. Even different linguistic contexts influence monolingual EF performance (Bice and Kroll, 2019). Furthermore, a recent large-scale study showed that 80% of their sample (*N* = 962) who self-classified as “monolingual” learned another language at some point (Castro et al., 2022), blurring the line between monolinguals and bilinguals even further. Attentional resources can also affect how people learn a second language, and this has implications for performance. In sum, it is critical that future studies examine the different bilingual experiences and contexts that interact with the various forms of attentional control in order to fully understand how bilingualism affects attention.

## Author Contributions

AC-F-Y conceived the idea. AC-F-Y, NC, and JG wrote the manuscript. All authors contributed to the article and approved the submitted version.

## Funding

The open-access publication fees for this article were covered by the Iowa State University Library.

## Conflict of Interest

The authors declare that the research was conducted in the absence of any commercial or financial relationships that could be construed as a potential conflict of interest.

## Publisher’s Note

All claims expressed in this article are solely those of the authors and do not necessarily represent those of their affiliated organizations, or those of the publisher, the editors and the reviewers. Any product that may be evaluated in this article, or claim that may be made by its manufacturer, is not guaranteed or endorsed by the publisher.

## Footnotes

^1^ www.braintrain.com
